# Variability in Intrahousehold Transmission of Ebola Virus, and Estimation of the Household Secondary Attack Rate

**DOI:** 10.1093/infdis/jix579

**Published:** 2017-11-11

**Authors:** Judith R Glynn, Hilary Bower, Sembia Johnson, Cecilia Turay, Daniel Sesay, Saidu H Mansaray, Osman Kamara, Alie Joshua Kamara, Mohammed S Bangura, Francesco Checchi

**Affiliations:** 1 Department of Infectious Disease Epidemiology, London School of Hygiene and Tropical Medicine, United Kingdom; 2 Save the Children, Freetown, Sierra Leone; 3 Save the Children, London, United Kingdom

**Keywords:** Ebola, transmission chains, intrahousehold, risk factors, secondary attack rate

## Abstract

Transmission between family members accounts for most Ebola virus transmission, but little is known about determinants of intrahousehold spread. From detailed exposure histories, intrahousehold transmission chains were created for 94 households of Ebola survivors in Sierra Leone: 109 (co-)primary cases gave rise to 317 subsequent cases (0–100% of those exposed). Larger households were more likely to have subsequent cases, and the proportion of household members affected depended on individual and household-level factors. More transmissions occurred from older than from younger cases, and from those with more severe disease. The estimated household secondary attack rate was 18%.

## Teaser

In a study of 94 Ebola-affected households, intrahousehold transmission was very variable, with more transmission from those with more severe disease and from older cases. The household secondary attack rate was 18%.

Although funerals and healthcare settings play an important role in the spread of Ebola virus, community transmission, mostly between family members, accounts for the majority of transmissions [1, 2]. Yet few studies have assessed transmission patterns within households, and what determines whether the infection is contained or spreads.

Studies of risk factors for Ebola virus disease (EVD) have focused on the exposure (infection is most likely following contact with dead bodies and bodily fluids [3–5]) and on the characteristics of the person exposed, with lower attack rates in children than adults [4, 6]. Less emphasis has been given to the characteristics of the source cases (other than the severity of disease that they had [7]) or of the households that may be associated with onward transmission, although behavioral and environmental factors are likely to influence exposure patterns [8]. A study of 27 households in Kikwit, Democratic Republic of Congo (DRC), in 1995 found no association between household characteristics and secondary attack rates [5].

Little is known about who transmits (except for a small number of reconstructed transmission chains; see, eg, [1, 2, 7]). In Yambuku, DRC, in 1976, the secondary attack rate was higher in closer relatives and from female source cases [9]. Two studies in Liberia found no difference in transmission by sex; in one [10], but not the other [8], there was less transmission from children than from adults.

In a study of 94 households of survivors, we have previously estimated exposure-specific and age-specific attack rates [4], risk factors for the acquisition of Ebola in young children [11], and the extent of asymptomatic infection [12]. In this analysis we reconstruct the likely within-household transmission chains to assess factors influencing transmission and who probably transmitted to whom; and estimate the household secondary attack rate, a key parameter for transmission modeling studies [13].

## METHODS

In July–September 2015, all survivors from Kerry Town Ebola Treatment Centre living in Western Area, Sierra Leone, and their household members, were invited for interview, as described elsewhere [4, 11]. Transmission chains were created for each household, based on the contact patterns described by the household members. We did not attempt to ascertain onset dates, given the time that had elapsed before interview, but all households only experienced 1 period with EVD cases. See Supplementary Figure 1 for definitions and Supplementary Figure 2 for an illustration of how the generations of transmission were derived.

Individual written informed consent was obtained from all participants (or their parents/guardians for those aged <18 years) before interview and sample collection. Permission for the study was granted by the Sierra Leone Ethics and Scientific Review Committee, and the Ethics Committee of the London School of Hygiene and Tropical Medicine.

### Statistical Analysis

We investigated whether any household transmission occurred (using logistic regression), and the proportion of household members infected (using generalized linear models), by characteristics of the primary case(s) and the household.

At the individual level, we assessed the characteristics of cases that were associated with transmission, including severity of illness, classified by symptoms while at home (wet symptoms [ie, diarrhea, vomiting, or bleeding] or only dry symptoms) and survival. We used negative binomial regression, because the number of subsequent cases was overdispersed, adjusted all analyses for household size (by including the number of people exposed as an exposure parameter), and allowed for household clustering using robust standard errors. All analyses used Stata version 14 software.

## RESULTS

Household members of 123 of the 151 Kerry Town Ebola survivors were living in Western Area and available for interview: 1 survivor refused and the others lived outside the area or were unavailable [4]. They lived in 94 households, which altogether contained 937 individuals, of whom 427 were infected with Ebola virus (including 10 asymptomatic infections and 11 undiagnosed symptomatic infections identified by serology [12], and 238 deaths). Four individuals with unclear causes of death are included as noncases. Household size varied from 1 to 27 people, with up to 21 EVD cases in a single household (see Supplementary Figure 3*A*).

Most households had a single primary case; 8 households had 2 cases, 1 had 3 cases, and 1 had 4 co-primaries (Supplementary Figure 3*B*). We excluded the 1 single-person household and 1 individual who already had EVD before joining a household with EVD cases. Two adults with unclear age were included in the largest adult age category (15–44 years).

### Household-Level Analyses

In the univariable analysis, household size and crowding, but no other available household-level measures, were associated with the risk of any subsequent cases occurring (Table 1). All 25 households in which a primary case died at home had subsequent cases. Onward transmission was more common from primaries with wet symptoms than dry symptoms, but 4 households with primary cases with dry symptoms had onward transmission. Subsequent cases were also more common in households with older primary cases.

**Table 1.** Characteristics of Households and of the Primary Case(s) in Relation to Spread of Ebola Virus in the Household

Household Characteristic	No. of Households	Any Secondary Cases			Mean Proportion Infected (95% CI)	*P* Value^b^
		No.	(%)	*P* Value^a^		
No. of people (excluding primaries)						
≤5	27	13	(48)	.003	0.24 (.11–.37)	<.0001
6–10	36	28	(78)		0.35 (.25–.44)	
≥11	30	26	(87)		0.42 (.31–.53)	
Mean age of exposed (excluding primaries)						
<17	31	19	(61.3)	.2	0.22 (.13–.31)	<.0001
17–20	31	23	(74.2)		0.36 (.25–.47)	
≥21	31	25	(80.1)		0.43 (.31–.56)	
Level of crowding						
>3/room	30	22	(73)	.04	0.40 (.29–.52)	<.0001
2–3/room	32	27	(84)		0.33 (.23–.43)	
<2/room	29	16	(55)		0.28 (.15–.41)	
Access to water						
Sometimes	18	11	(61)	.2	0.28 (.11–.44)	.2
Most days	29	19	(66)		0.33 (.22–.45)	
Every day	45	36	(80)		0.36 (.27–.45)	
Access to soap						
Sometimes	26	19	(73)	.8	0.29 (.19–.39)	.4
Most days	18	14	(78)		0.46 (.29–.62)	
Every day	48	33	(69)		0.32 (.22–.41)	
Setting						
Rural	23	19	(83)	.2	0.30 (.21–.40)	.05
Urban	70	48	(69)		0.35 (.27–.43)	
Healthcare worker in household						
No	78	56	(72)	.9	0.33 (.27–.40)	.5
Yes	15	11	(73)		0.38 (.17–.59)	
Persons moved out of household						
No	78	54	(69)	.2	0.34 (.27–.41)	.6
Yes	15	13	(87)		0.33 (.19–.47)	
Period						
November–mid-December	47	34	(72)	.9	0.36 (.27–.45)	.001
Mid-December to March	46	33	(72)		0.32 (.23–.41)	
Primary^c^						
Illness while at home						
Dry symptoms	13	4	(31)	<.001	0.09 (0–.21)	<.0001
Symptoms unknown	2	2	(100)		0.25 (.0–1.0)	
Wet symptoms	39	24	(62)		0.31 (.21–.41)	
Died, location unknown	14	12	(86)		0.36 (.19–.52)	
Died at home	25	25	(100)		0.51 (.40–.62)	
No. of primary cases						
1	81	59	(73)	.7	0.36 (.29–.43)	.001
>1	12	8	(67)		0.21 (.072–.35)	
Child (<15 y)						
No	86	62	(73)	.97	0.34 (.28–.41)	.02
Yes	7	5	(71)		0.28 (.0–.60)	
Aged ≥45 y						
No	61	38	(62)	.004	0.27 (.19–.34)	<.0001
Yes	32	29	(91)		0.47 (.38–.57)	
Household head						
No	55	41	(75)	.5	0.36 (.28–.44)	.8
Yes	38	26	(68)		0.31 (.21–.41)	
Male						
No	43	33	(77)	.3	0.39 (.29–.49)	.01
Yes	50	34	(68)		0.30 (.21–.38)	

Mean proportion infected defined as the number of nonprimary cases divided by the number exposed (ie household members excluding the primary cases). Missing data: crowding for 2, water and soap for 1.

a χ^2^ test.

b From generalized linear model. Given as *P* value for trend across categories if >2 categories.

c Where there was >1 primary, the variables are coded as present if at least 1 primary had that characteristic, and the most severe manifestation of disease was selected.

In multivariable analysis the associations with crowding and older primaries were lost, and the only factors influencing the risk of any secondary transmission were the number exposed and the severity of illness of the primary case(s). The crude odds ratio (OR) for the association with number exposed (1.3; 95% confidence interval [CI], 1.1–1.5, for each additional person exposed) was only slightly reduced by adjusting for the severity of illness of the primary case (OR, 1.2; 95% CI, 1.0–1.5).

Several factors were associated with the proportion of household members infected (Table 1). After adjustment in the generalized linear model, the proportion was higher in more crowded households, households with older people, and if the primary was ≥45 years old, head of household, female, or had more severe illness (Supplementary Table 1). Households that included a healthcare worker or were infected later in the epidemic had a lower proportion infected.

### Individual-Level Analyses

A third of those infected with Ebola virus transmitted to someone else in the household (139/425 [33%]; Table 2). More than half of those who transmitted (55%) transmitted to 2 or more people (Supplementary Figure 4). Of those who transmitted, 108 died, 29 became survivors from treatment centers, 1 was an unrecognized symptomatic case, and 1 was an asymptomatic household member with positive serology (who was the most likely source for her 1-week-old baby).

**Table 2.** Factors Influencing Onward Transmission of Ebola Virus Within the Household by Characteristics of the Source Cases, Sierra Leone 2014–2015

Characteristics of Source	Total No. Infected	No. (%) Transmitting	No. of Persons Infected by Each Case														
			0	(%)	1	(%)	2	(%)	≥3	(%)	Mean	Crude IRR^a^	(95% CI)	*P* Value	Adjusted IRR^b^	(95% CI)	*P* Value
Total	425	139 (32.7)	286	(67.3)	63	(14.8)	36	(8.5)	40	(9.4)	0.7						
Sex of source																	
Female	252	88 (34.9)	164	(65.1)	38	(15.1)	27	(10.7)	23	(9.1)	0.8	1					
Male	173	51 (29.5)	122	(70.5)	25	(14.5)	9	(5.2)	17	(9.8)	0.7	0.94	(.65–1.4)	.74			
Age of source																	
<5 y	51	6 (11.8)	45	(88.2)	5	(9.8)	0	(0.0)	1	(2.0)	0.2	1			1		
5–14 y	64	12 (18.8)	52	(81.3)	10	(15.6)	2	(3.1)	0	(0.0)	0.2	1.7	(.05–5.6)		2.6	(1.1–5.8)	
15–44 y	229	75 (32.8)	154	(67.2)	35	(15.3)	24	(10.5)	16	(7.0)	0.7	5.2	(1.8–15.0)		4.2	(2.1–8.4)	
≥45 y	81	46 (56.8)	35	(43.2)	13	(16.0)	10	(12.3)	23	(28.4)	1.7	12.6	(4.2–37.8)	<.001	4.8	(2.3–10.1)	<.001
Source was primary case																	
No	317	67 (21.1)	250	(78.9)	38	(12.0)	19	(6.0)	10	(3.2)	0.4	1			1		
Yes	108	72 (66.7)	36	(33.3)	25	(23.1)	17	(15.7)	30	(27.8)	1.8	6.7	(4.8–9.4)	<.001	3.0	(2.0–4.5)	<.001
Severity of illness of source at home																	
Dry: survived	65	8 (12.3)	57	(87.7)	7	(10.8)	1	(1.5)	0	(0.0)	0.1	1			1		
Dry: died away from home	5	1 (20.0)	4	(80.0)	0	(0.0)	0	(0.0)	1	(20.0)	0.8	7.4	(.74–74.8)		3.7	(.61–22.7)	
Unknown symptoms: survived	38	2 (5.3)	36	(94.7)	2	(5.3)	0	(0.0)	0	(0.0)	0.1	0.40	(.09–1.7)		0.46	(.10–2.2)	
Unknown symptoms: died away	15	3 (20.0)	12	(80.0)	2	(13.3)	0	(0.0)	1	(6.7)	0.3	1.7	(.35–8.3)		1.8	(.59–5.5)	
Wet: survived	85	21 (24.7)	64	(75.3)	12	(14.1)	6	(7.1)	3	(3.5)	0.4	3.3	(1.6–6.9)		2.8	(1.3--5.7)	
Wet: died away from home	37	30 (81.1)	7	(18.9)	10	(27.0)	6	(16.2)	14	(37.8)	2.1	16.6	(8.5–32.3)		8.6	(4.4–17.1)	
Died location unknown	138	36 (26.1)	102	(73.9)	18	(13.0)	14	(10.1)	4	(2.9)	0.5	2.3	(1.04–5.2)		2.2	(1.1–4.7)	
Died at home	42	38 (90.5)	4	(9.5)	12	(28.6)	9	(21.4)	17	(40.5)	2.7	18.4	(9.9–34.2)	<.001	9.8	(4.8–20.1)	<.001
Occupation of source																	
Not healthcare worker	414	131 (31.6)	283	(68.4)	61	(14.7)	35	(8.5)	35	(8.5)	0.7	1					
Health worker	11	8 (72.7)	3	(27.3)	2	(18.2)	1	(9.1)	5	(45.5)	3.0	4.6	(2.6–8.1)	<.001			
Source’s position in household																	
Household member	360	105 (29.2)	255	(70.8)	50	(13.9)	30	(8.3)	25	(6.9)	0.6	1					
Household head	65	34 (52.3)	31	(47.7)	13	(20.0)	6	(9.2)	15	(23.1)	1.5	3.5	(2.3–5.3)	<.001			

Abbreviations: CI, confidence interval; IRR, incidence rate ratio.

a IRR adjusted for cluster and household size.

b IRR adjusted for cluster, household size and all variables in the model. *P* values from likelihood ratio test.

Factors associated with onward transmission are shown in Table 2. After adjusting just for household size and household clustering, the likelihood of onward transmission was similar for males and females and was higher from older cases, from primary cases, from those with more severe disease, and from healthcare workers and household heads.

In the multivariable analysis, the associations with household head and healthcare workers were lost. The associations with age and severity of illness were reduced but were still strong. To see whether the excess risk of transmission from those who died was entirely due to contact with the corpse, further analysis excluded those dying at home: The adjusted incidence rate ratio for transmission was 3.1 (95% CI, 1.9–5.2) comparing wet cases who died away from the home to wet cases who survived.

Supplementary Table 2 shows the characteristics of the likely sources for all nonprimary cases in the households. There was little evidence for assortative or disassortative transmission between the sexes, but there was disassortative transmission by age: Children were infected more by those aged 15–44 and less by those aged ≥45 than would be expected by chance. The households had up to 5 generations of transmission (Supplementary Figure 3*B*). The proportion of cases infected as secondary cases, rather than in subsequent generations, was lower among young children (Supplementary Table 2).

### Household Secondary Attack Rate

The 109 primary/co-primary cases gave rise to 201 secondary cases (Supplementary Table 2, including 7 with asymptomatic infections) among 827 exposed household members, giving a secondary attack rate of 24% and reproduction number in the first generation of intrahousehold transmission of 1.8. The overall proportion of household members infected (household attack rate), excluding the primary cases, was 38% (317/827). As survivor households tend to have more cases (increasing the chance that some survived), these attack rates are likely to be overestimates. The case fatality rate for this epidemic was around two-thirds so we can adjust for this bias by assuming that for each household with only surviving cases, 2 households with the same number of only fatal cases were missed, and that these households were the same size as the households with only surviving cases. This adjustment gives an estimated household secondary attack rate of 18%, reproduction number of 1.2, and household attack rate of 28%. With this adjustment, the median household size is reduced from 12 to 8, and the case fatality rate increased from 56% to 62%.

## DISCUSSION

The households in this study had a variety of experiences, from those with a single EVD case and no subsequent spread to those with all members affected. Key drivers of household transmission included severity of illness and increasing age of the source. Household size was an important determinant of initial spread, but did not influence the total proportion infected once adjusted for other factors. Those with only dry symptoms were less likely to transmit, but one-third of households with primary cases with dry symptoms had subsequent cases.

The association with age of the source was not fully explained by severity of illness, or the fact that primary cases (who have more susceptibles to transmit to) tend to be older [4], or by the tendency for young children to be infected in later generations (Supplementary Table 2). Possible explanations include young children being cared for by the parent who was also the source of their infection, and the respect given to older people, leading to people ministering to them. Our study contradicts inferences of a modeling study, which predicted more transmission from children [14]. Lower transmission from children was also found in transmission chains in Liberia [10], with no difference by age found in a study based on contact tracing data [8].

The association with crowding was expected, but the lack of association with sanitation is surprising. Few households moved people out, and where this did happen it may have been too late to avert exposure. We found less transmission later in the epidemic, suggesting improved knowledge of what to do, and helped by a greater availability of Ebola care beds; having a healthcare worker in the household also reduced transmission. Associations with more transmission if the primary case was head of the household or female were not supported in the individual-level analysis, after adjustment for other factors.

The household secondary attack rate was high. At 18%, it is closer to reports from Kikwit, DRC (16%) [5] and Nzara/Yambio, Sudan (13%) [15] than to Yambuku (8%) [9] or the 6% estimated for Sierra Leone (which relied on matching names and addresses from case report forms) [13], or 4% in Liberia (based on shared surnames and communities in contact tracing data) [8].

This analysis relied on histories collected in interviews 4–9 months after the events. By interviewing the household members as a group, we hoped to maximize recall. We did not attempt to record dates of onset, so have not used the serial interval but have based the transmission chains on the reported order of events, and types of contact, favoring higher levels of exposure where multiple sources were possible. Some misclassification is likely and our method may have contributed to the association with severity of illness in the individual-level analysis, but not to the associations with severity of illness in the household analysis or to the higher transmission from those who died away from the home than from those who survived. This last finding, which is in contrast to findings in Liberia and Guinea [7], may be explained by higher viral loads while at home in those who subsequently died. The association of transmission with severity of illness underscores the importance of early identification and isolation of cases.

## CONCLUSIONS

Initial spread in the household was more likely in larger households, and cases with more severe disease, particularly deaths, and older cases had more onward transmissions. Our estimate for reproduction number in the first generation of household transmission of only a little above 1, and the reduced proportion of household members affected later in the epidemic, suggest that it should be feasible to curtail intrahousehold transmission more rapidly.

## Supplementary Data

Supplementary materials are available at *The Journal of Infectious Diseases* online. Consisting of data provided by the authors to benefit the reader, the posted materials are not copyedited and are the sole responsibility of the authors, so questions or comments should be addressed to the corresponding author.

Glynn Ebola Appendix

## Notes

Presented in part: Eighth International Symposium on Filoviruses/58th ITM Colloquium, Antwerp, Belgium, 12�15 September 2016. Abstract 4.

## Notes

***Acknowledgments.*** We thank all the participants for the time and thought they gave to the study, and Save the Children country office for their support. We also thank the Sierra Leone Ministries of Health and Sanitation and of Social Welfare, Gender and Children’s Affairs for their permission to carry out the study and support throughout. We thank Professor Neal Alexander for statistical advice.

***Financial support.*** The study was supported by grants from Save the Children internal funds and the Wellcome Trust’s Enhancing Research Activity in Epidemic Situations (ERAES) program (grant number 107779/Z/15/Z).

***Potential conflicts of interest.*** Save the Children International operated the Kerry Town Ebola Treatment Centre during the period under study and employed the field team members. One author (F. C.) was employed by Save the Children UK and was involved in commissioning the study and interpreting findings. All other authors report no potential conflicts. All authors have submitted the ICMJE Form for Disclosure of Potential Conflicts of Interest. Conflicts that the editors consider relevant to the content of the manuscript have been disclosed.
