# Supplementary material for: Variability in Intrahousehold Transmission of Ebola Virus, and Estimation of the Household Secondary Attack Rate
Source: J Infect Dis. 2017 Nov 11;217(2):232–7. doi: 10.1093/infdis/jix579 (PMC5853870; doi:10.1093/infdis/jix579)

**Appendix**

**Variability in intra-household transmission of Ebola virus,**

**and estimation of the household secondary attack rate**

Judith R Glynn, Hilary Bower, Sembia Johnson, Cecilia Turay, Daniel Sesay, Saidu H. Mansaray, Osman Kamara, Alie Joshua Kamara, Mohammed S Bangura, Francesco Checchi

**Table S1. Risk factors influencing proportion infected with Ebola virus in survivor households, Sierra Leone 2014-2015. Results from generalised linear model.**

|  |  | **Crude** | | |  | **Adjusted^a^** |  |
| --- | --- | --- | --- | --- | --- | --- | --- |
| **Household** | | OR | CI | p | OR | CI | P |
| No. of people | |  |  |  |  |  |  |
|  |  | 1.1 | 1.0-1.1 | <0.0001 | 1.0 | 0.97-1.04 | 0.9 |
| Mean age of exposed | |  |  |  |  |  |  |
|  | <17 | ref |  | <0.0001 | ref |  | <0.0001 |
|  | 17-20 | 1.9 | 1.3-2.8 |  | 2.4 | 1.5-3.8 |  |
|  | ≥21 | 3.0 | 2.1-4.4 |  | 5.3 | 3.1-0.2 |  |
| Level of crowding | |  |  |  |  |  |  |
|  | Decreasing crowding^b^ | 0.70 | 0.58-0.85 | <0.0001 | 0.67 | 0.53-0.86 | 0.002 |
| Healthcare worker in household | | |  |  |  |  |  |
|  | vs none | 1.1 | 0.80-1.6 | 0.5 | 0.51 | 0.33-0.79 | 0.002 |
| Period |  |  |  |  |  |  |  |
|  | Late vs early | 0.62 | 0.47-0.82 | 0.001 | 0.43 | 0.29-0.64 | <0.0001 |
|  |  |  |  |  |  |  |  |
| **Primary** |  |  |  |  |  |  |  |
| Illness while at home | |  |  |  |  |  |  |
|  | Dry symptoms | ref |  | <0.0001 | ref |  | <0.0001 |
|  | Symptoms unknown | 2.9 | 0.75-11.1 |  | 0.85 | 0.20-3.5 |  |
|  | Wet symptoms | 4.8 | 2.2-10.3 |  | 2.8 | 1.2-6.5 |  |
|  | Died, location unknown | 3.8 | 1.7-8.7 |  | 4.8 | 1.9-12.0 |  |
|  | Died at home | 9.6 | 4.4-20.6 |  | 5.0 | 2.2-11.8 |  |
| Aged >=45 | |  |  |  |  |  |  |
|  | vs <45 | 1.8 | 1.4-2.4 | <0.0001 | 2.0 | 1.4-2.0 | <0.0001 |
| Head of household | | | | | | | |
|  | vs not | 0.95 | 0.71-1.3 | 0.8 | 3.3 | 2.0-5.5 | <0.0001 |
| Male primary | |  |  |  |  |  |  |
|  | vs only female | 0.70 | 0.53-0.92 | 0.01 | 0.43 | 0.29-0.64 | <0.0001 |

*^a^ Adjusted for all the factors shown in the table . Factors were included if they had an association with the outcome in the adjusted model at the 5% level. The number of people in the household was included because it had a large effect on the risk of any secondary spread, although it was not associated with the proportion infected, as seen here.*

*^b^Linear trend across categories*

**Table S2: Characteristics of likely source cases for individuals infected with Ebola virus in their households, Sierra Leone, 2014-15**

|  | **Characteristics of non-primary cases** | | | | | | | | | | | | | | | |
| --- | --- | --- | --- | --- | --- | --- | --- | --- | --- | --- | --- | --- | --- | --- | --- | --- |
| **Likely sources of transmission** | **Female** | ***%*** | **Male** | ***%*** | ***P*^a^** | **<5y** | ***%*** | **5-14y** | ***%*** | **15-44y** | ***%*** | **≥45y** | ***%*** | ***P*^a^** | **Total** | ***%*** |
|  | 189 | *100.0* | 118 | *100.0* |  | 48 | *100.0* | 58 | *100.0* | 154 | *100.0* | 47 | *100.0* |  | 307 |  |
| **Sex of source** |  |  |  |  |  |  |  |  |  |  |  |  |  |  |  |  |
| Female | 121 | *64.0* | 71 | *60.2* |  | 34 | *70.8* | 42 | *72.4* | 85 | *55.2* | 31 | *66.0* |  | 192 | *62.5* |
| Male | 68 | *36.0* | 47 | *39.8* | 0.50 | 14 | *29.2* | 16 | *27.6* | 69 | *44.8* | 16 | *34.0* | 0.06 | 115 | *37.5* |
| **Age of source** |  |  |  |  |  |  |  |  |  |  |  |  |  |  |  |  |
| <5 years | 7 | *3.7* | 1 | *0.90* |  | 0 | *0.0* | 2 | *3.4* | 3 | *1.9* | 3 | *6.4* |  | 8 | *2.6* |
| 5-14 years | 7 | *3.7* | 7 | *5.9* |  | 4 | *8.3* | 3 | *5.2* | 6 | *3.9* | 1 | *2.1* |  | 14 | *4.6* |
| 15-44 years | 89 | *47.1* | 60 | *50.8* |  | 27 | *56.3* | 36 | *62.1* | 68 | *44.2* | 18 | *38.3* |  | 149 | *48.5* |
| ≥45 years | 86 | *45.5* | 50 | *42.4* | 0.34 | 17 | *35.4* | 17 | *29.3* | 77 | *50.0* | 25 | *53.2* | 0.04 | 136 | *44.3* |
| **Relationship of source to infected person** |  |  |  |  |  |  |  |  |  |  |  |  |  |  |  |  |
| Spouse | 32 | *16.9* | 17 | *14.4* |  | 0 | *0.0* | 0 | *0.0* | 32 | *20.8* | 17 | *36.2* |  | 49 | *16.0* |
| Child | 13 | *6.9* | 2 | *1.7* |  | 0 | *0.0* | 0 | *0.0* | 8 | *5.2* | 7 | *14.9* |  | 15 | *4.9* |
| Mother | 55 | *29.1* | 26 | *22.0* |  | 22 | *45.8* | 25 | *43.1* | 32 | *20.8* | 2 | *4.3* |  | 81 | *26.4* |
| Father | 15 | *7.9* | 19 | *16.1* |  | 10 | *20.8* | 6 | *10.3* | 17 | *11.0* | 1 | *2.1* |  | 34 | *11.1* |
| Sibling | 24 | *12.7* | 24 | *20.3* |  | 3 | *6.3* | 7 | *12.1* | 31 | *20.1* | 7 | *14.9* |  | 48 | *15.6* |
| Grandchild | 4 | *2.1* | 1 | *0.0* |  | 0 | *0.0* | 0 | *0.0* | 0 | *0.0* | 5 | *10.6* |  | 5 | *1.6* |
| Grandparent | 8 | *4.2* | 8 | *7.6* |  | 8 | *16.7* | 3 | *5.2* | 5 | *3.2* | 0 | *0.0* |  | 16 | *5.2* |
| In-laws | 9 | *4.8* | 1 | *0.85* |  | 0 | *0.0* | 1 | *1.7* | 5 | *3.2* | 4 | *8.5* |  | 10 | *3.3* |
| Niece/nephew | 3 | *1.6* | 3 | *2.5* |  | 0 | *0.0* | 1 | *1.7* | 4 | *2.6* | 1 | *2.1* |  | 6 | *2.0* |
| Cousin | 4 | *2.1* | 2 | *1.7* |  | 0 | *0.0* | 3 | *5.2* | 3 | *1.9* | 0 | *0.0* |  | 6 | *2.0* |
| Aunt/Uncle | 9 | *4.8* | 7 | *5.9* |  | 2 | *4.2* | 10 | *17.2* | 4 | *2.6* | 0 | *0.0* |  | 16 | *5.2* |
| Other | 13 | *6.9* | 8 | *6.8* | n/a | 3 | *6.3* | 2 | *3.4* | 13 | *8.4* | 3 | *6.4* | n/a | 21 | *6.8* |
| **Position of source in household** |  |  |  |  |  |  |  |  |  |  |  |  |  |  |  |  |
| Household member | 123 | *65.1* | 86 | *72.9* |  | 38 | *79.2* | 43 | *74.1* | 91 | *59.1* | 37 | *78.7* |  | 209 | *68.1* |
| Household head | 66 | *34.9* | 32 | *27.1* | 0.15 | 10 | *20.8* | 15 | *25.9* | 63 | *40.9* | 10 | *21.3* | 0.01 | 98 | *31.9* |
| **Source was health care worker** |  |  |  |  |  |  |  |  |  |  |  |  |  |  |  |  |
| No | 166 | *87.8* | 108 | *91.5* |  | 46 | *95.8* | 57 | *98.3* | 133 | *86.4* | 38 | *80.9* |  | 274 | *89.3* |
| Yes | 23 | *12.2* | 10 | *8.5* | 0.31 | 2 | *4.2* | 1 | *1.7* | 21 | *13.6* | 9 | *19.1* | 0.004 | 33 | *10.7* |
| **Severity of illness of source at home** |  |  |  |  |  |  |  |  |  |  |  |  |  |  |  |  |
| Dry symptoms: survived | 4 | *2.1* | 5 | *4.2* |  | 2 | *4.2* | 3 | *5.2* | 4 | *2.6* | 0 | *0.0* |  | 9 | *2.9* |
| Dry symptoms: died away from home | 3 | *1.6* | 1 | *0.85* |  | 1 | *2.1* | 2 | *3.4* | 0 | *0.0* | 1 | *2.1* |  | 4 | *1.3* |
| Unknown symptoms: survived | 1 | *0.53* | 1 | *0.85* |  | 1 | *2.1* | 1 | *1.7* | 0 | *0.0* | 0 | *0.0* |  | 2 | *0.65* |
| Unknown symptoms: died away from home | 3 | *1.6* | 2 | *1.7* |  | 2 | *4.2* | 0 | *0.0* | 2 | *1.3* | 1 | *2.1* |  | 5 | *1.6* |
| Wet symptoms: survived | 23 | *12.2* | 12 | *10.2* |  | 11 | *22.9* | 13 | *22.4* | 11 | *7.1* | 0 | *0.0* |  | 35 | *11.4* |
| Wet symptoms: died away from home | 49 | *25.9* | 31 | *26.3* |  | 9 | *18.8* | 11 | *19.0* | 44 | *28.6* | 16 | *34.0* |  | 80 | *26.1* |
| Died symptoms: location unknown | 32 | *16.9* | 27 | *22.9* |  | 10 | *20.8* | 16 | *27.6* | 24 | *15.6* | 9 | *19.1* |  | 59 | *19.2* |
| Died at home | 74 | *39.2* | 39 | *33.1* | 0.78 | 12 | *25.0* | 12 | *20.7* | 69 | *44.8* | 20 | *42.6* |  | 113 | *36.8* |
| **Position of source in transmission chain** |  |  |  |  |  |  |  |  |  |  |  |  |  |  |  |  |
| Primary case | 133 | *68.5* | 68 | *56.2* |  | 21 | *43.8* | 31 | *53.4* | 113 | *69.8* | 36 | *76.6* |  | 201 | *63.8* |
| Secondary case | 45 | *23.2* | 42 | *34.7* |  | 17 | *35.4* | 24 | *41.4* | 37 | *22.8* | 9 | *19.1* |  | 87 | *27.6* |
| Tertiary case | 12 | *6.2* | 9 | *7.4* |  | 6 | *12.5* | 3 | *5.2* | 11 | *6.8* | 1 | *2.1* |  | 21 | *6.7* |
| Quaternary or more | 4 | *2.1* | 2 | *1.7* | 0.12 | 4 | *8.3* | 0 | *0.0* | 1 | *0.62* | 1 | *2.1* | <0.001^b^ | 6 | *1.9* |

**Notes:** Missing data in subsequent cases: 8 subsequent cases with multiple possible sources of infection included only in analysis of position of source in transmission chain; 2 no information on source of infection.

Of the 54 who died away from home whose place of death was known, 49 died in Ebola Treatment or Holding Centres, one died with a traditional healer, one was asked to leave the home and died on a bus, and three moved to other households (two to the same household).

^a^ Chi^2^ or Fisher's exact tests. ^b^ Chi^2^ using linear regression.

n/a = not applicable (numbers in subgroups too small)

*.*

**Figure S1: Definitions used**

**Household**: People eating from the same pot who were resident while members of the household had EVD. Includes people who had not been resident in the household before Ebola occurred (e.g. external family members who joined the household to care for sufferers).

**Case:** EVD survivors from treatment centres; those reported by the family to have died of Ebola or who died and had symptoms fitting the case definition of EVD; and people with positive IgG to Ebola (symptomatic or asymptomatic).

**Primary case:** The first person with symptoms in the household. For some households more than one person was described as having symptoms at the same time, usually following a common external exposure. They have been treated as co-primaries.

**Subsequent case:** Cases in the household who were not primary cases.

**Wet case:** Case of EVD with diarrhoea, vomiting or bleeding.

**Dry case:** Case of EVD without diarrhoea, vomiting or bleeding.

**Levels of exposure:** This 8-level scale was created a priori based on literature and discussion with Ebola treatment centre staff. The highest level was contact with the body of those who died of Ebola; then direct contact with body fluids of those with Ebola, including breast feeding, then direct contact with “wet” cases; then direct contact with dry cases; indirect contact with a wet case (eg with washed clothes); indirect contact with a dry case; minimal contact (eg shared utensils); no contact known.

**Transmission/source of infection:** The most likely routes of transmission were estimated from the contact histories. For example, if someone was reported to have had a direct physical contact with a case while they were ill, this case was taken as the likely source for that transmission. If more than one contact was reported we selected the one more likely to transmit, i.e. the one with the greatest degree of exposure, based on the levels of exposure and contact patterns described.

**Generation of transmission:** Primary cases are the first generation. Secondary cases are those thought to have been infected by the primary cases; tertiary cases those infected by the secondary cases, and so on.

**Household secondary attack rate:** Secondary cases/(household members - primary cases)

**Household attack rate:** Subsequent cases/(household members - primary cases)

**Reproduction number (R) in the first generation of transmission:** secondary cases/primary cases in the household

**Figure S2: Illustration of reconstruction of transmission chains in a large and complex household**

**Figure S3. Experience of Ebola in 94 households of Ebola survivors, Sierra Leone 2014-15 (a) Showing deaths, cases of EVD and asymptomatic infections (b) Showing generations of transmission. Each column represents a different household.**

**
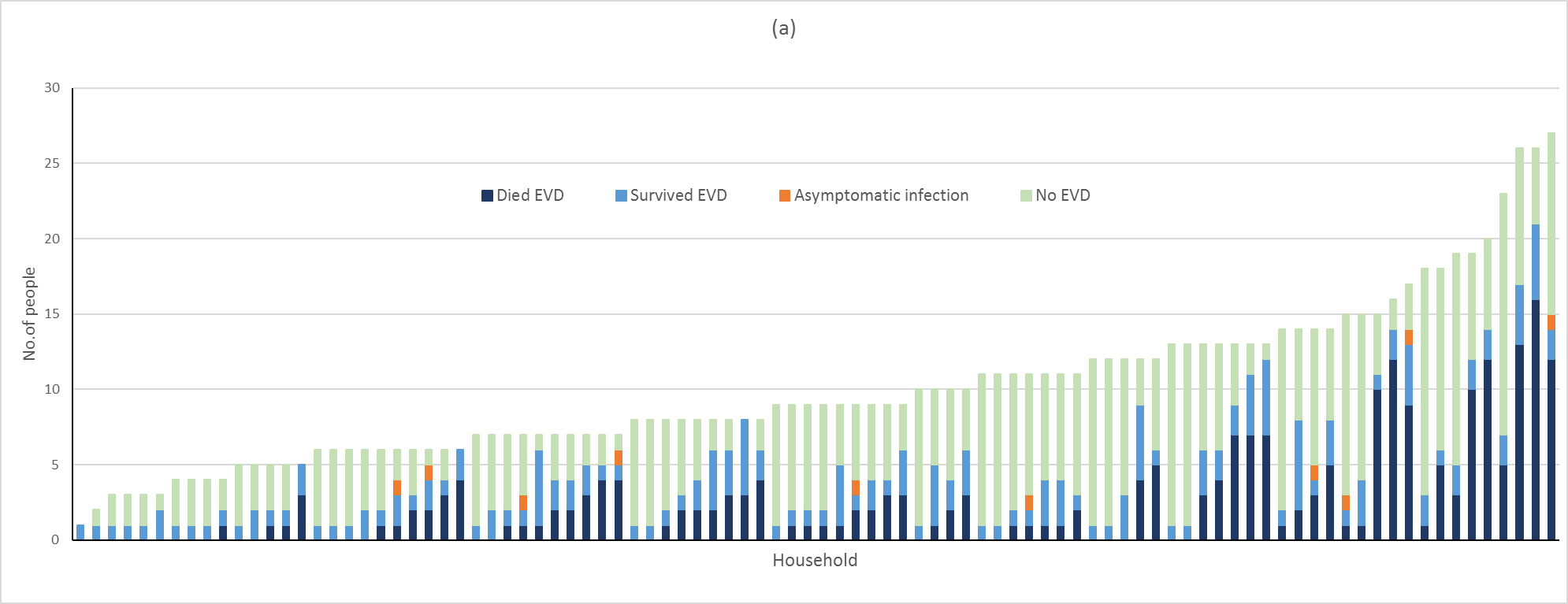
**

**
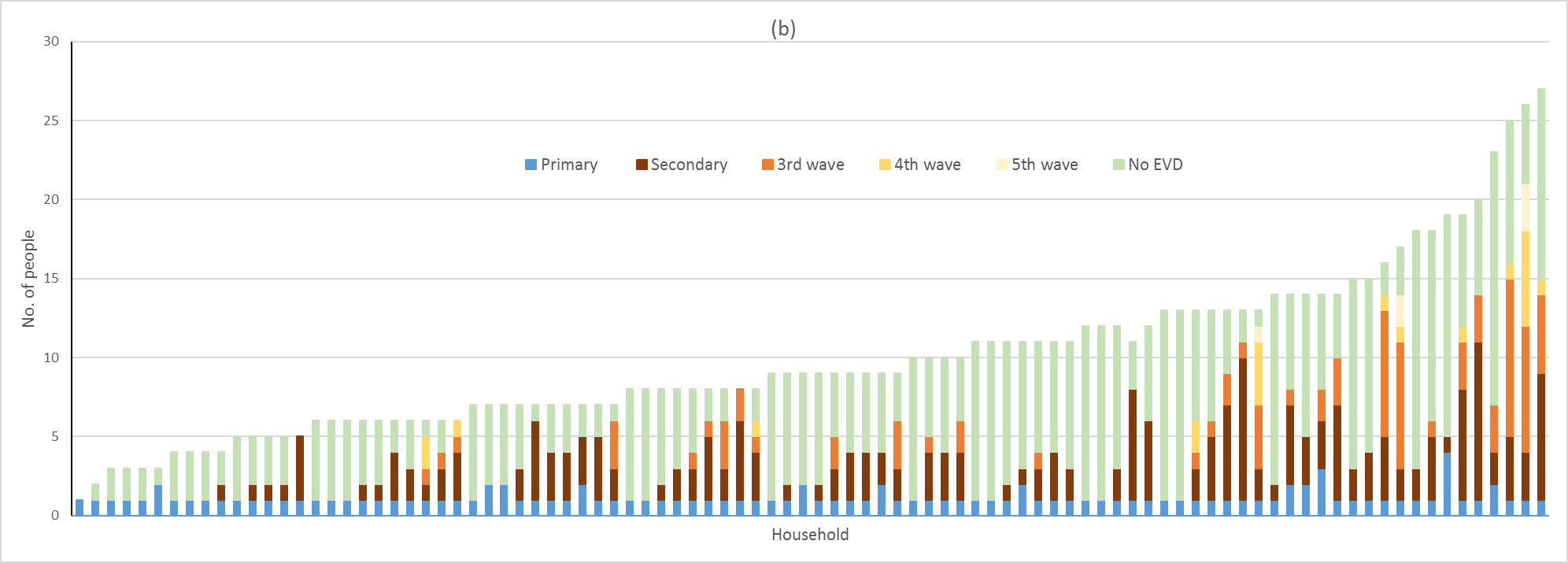
**

**Figure S4. Number of intra-household transmissions per EVD case in households of Ebola survivors, Sierra Leone 2014-15**


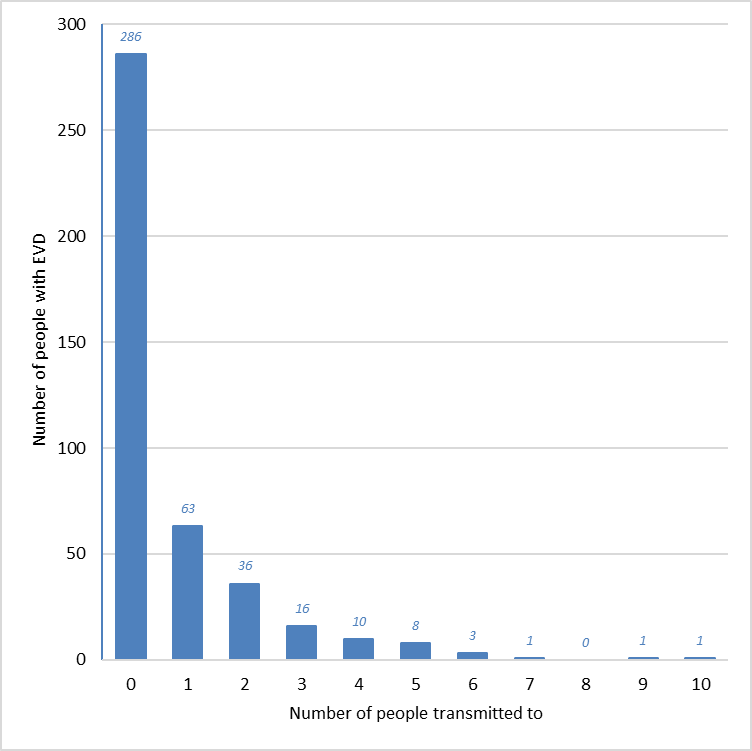

Supplement: Glynn Ebola Appendix [file jix579_suppl_glynn_ebola_appendix.docx]
